# Electronically delivered, multicomponent intervention to reduce unnecessary antibiotic prescribing for respiratory infections in primary care: a cluster randomised trial using electronic health records—REDUCE Trial study original protocol

**DOI:** 10.1136/bmjopen-2015-010892

**Published:** 2016-08-04

**Authors:** Dorota Juszczyk, Judith Charlton, Lisa McDermott, Jamie Soames, Kirin Sultana, Mark Ashworth, Robin Fox, Alastair D Hay, Paul Little, Michael V Moore, Lucy Yardley, A Toby Prevost, Martin C Gulliford

**Affiliations:** 1 Department of Primary Care and Public Health Sciences, King's College London, London, UK; 2 Clinical Practice Research Datalink, Medicines and Healthcare Products Regulatory Agency, London, UK; 3 Bicester Health Centre, Bicester, UK; 4 Centre for Academic Primary Care, School of Social and Community Medicine, University of Bristol, Bristol, UK; 5 Primary Care Research Group, University of Southampton, Southampton, UK; 6 Department of Psychology, University of Southampton, Southampton, UK

**Keywords:** Drug Resistance, Anti-Bacterial Agents, Respiratory Tract Infections, Electronic Health Records, Primary Health Care, Random Allocation

## Abstract

**Introduction:**

Respiratory tract infections (RTIs) account for about 60% of antibiotics prescribed in primary care. This study aims to test the effectiveness, in a cluster randomised controlled trial, of electronically delivered, multicomponent interventions to reduce unnecessary antibiotic prescribing when patients consult for RTIs in primary care. The research will specifically evaluate the effectiveness of feeding back electronic health records (EHRs) data to general practices.

**Methods and analysis:**

2-arm cluster randomised trial using the EHRs of the Clinical Practice Research Datalink (CPRD). General practices in England, Scotland, Wales and Northern Ireland are being recruited and the general population of all ages represents the target population. Control trial arm practices will continue with usual care. Practices in the intervention arm will receive complex multicomponent interventions, delivered remotely to information systems, including (1) feedback of each practice's antibiotic prescribing through monthly antibiotic prescribing reports estimated from CPRD data; (2) delivery of educational and decision support tools; (3) a webinar to explain and promote effective usage of the intervention. The intervention will continue for 12 months. Outcomes will be evaluated from CPRD EHRs. The primary outcome will be the number of antibiotic prescriptions for RTIs per 1000 patient years. Secondary outcomes will be: the RTI consultation rate; the proportion of consultations for RTI with an antibiotic prescribed; subgroups of age; different categories of RTI and quartiles of intervention usage. There will be more than 80% power to detect an absolute reduction in antibiotic prescription for RTI of 12 per 1000 registered patient years. Total healthcare usage will be estimated from CPRD data and compared between trial arms.

**Ethics and dissemination:**

Trial protocol was approved by the National Research Ethics Service Committee (14/LO/1730). The pragmatic design of the trial will enable subsequent translation of effective interventions at scale in order to achieve population impact.

**Trial registration number:**

ISRCTN95232781; Pre-results.

**Strengths and limitations of this study**

- This intervention will use electronic health records as means to inform, deliver and evaluate the effectiveness of a cluster trial to support antibiotic prescribing. This intervention, if effective, could be easily translated into routine practice settings.
- If successful, this study could help reduce antibiotic resistance—a growing problem that transcends national boundaries
- Although behavioural theory and qualitative research were used to enhance the effectiveness of intervention design, it was not possible to include all identified factors without creating an intervention which would be too complex and difficult to use.
- It is possible that the intervention will have a smaller effect than expected as problems with implementation might be encountered (eg, low adherence to electronic prompts). This will be examined during process evaluation.
- Initiatives to influence antibiotic prescribing both locally and nationally could influence the results of the current trial if these external influences contributed to optimal performance improvement across both trial arms.

## Background

Respiratory tract infections (RTIs), including cough, acute bronchitis, common colds, otitis media, sinusitis and sore throat (including laryngitis, pharyngitis and tonsillitis) are among most common presentations in primary care.^1^ ^2^ A majority of these infections are self-limiting^3–5^ and the UK guidance recommends no antibiotic strategy or a delayed antibiotic prescription for otherwise healthy adults,^2^ but ∼50% of patients who present with a RTI are prescribed an antibiotic.^6^ This overprescribing has negative consequences for the patients and for the wider public. Antibiotics can be associated with a number of possible unpleasant side effects for patients such as thrush or diarrhoea, and occasionally they can cause severe allergic reactions.^7^ Inappropriate prescribing increases the perception that antibiotics are an effective treatment for self-limiting infections increasing the likelihood of consultation for a similar condition in the future as patients believe that antibiotics are effective in these circumstances.^8^

Overusage of antibiotics in primary care also contributes to the emergence of antimicrobial drug resistance, increasing the risk of infections that may be very difficult to treat in the local community as well as for individual patients. Research evidence suggests that patients prescribed antibiotics for respiratory or urinary tract infection in primary care might develop bacterial resistance for up to 12 months.^9^ Recent analyses of data from Clinical Practice Research Datalink (CPRD) suggest an overall prescribing proportion of between 50% and 60% for RTIs, with 70% of episodes of otitis media and 90% of episodes of sinusitis resulting in antibiotic prescription.^1^ ^6^ Such rates of prescribing suggest that nearly all general practices are currently prescribing antibiotics at rates that are ‘way off the mark’^10^ in the context of good practice recommendations, which advise that most RTIs can be managed without the prescription of antibiotics.^2^ Although there are no available guidelines on safe level of antibiotic prescribing for RTIs, the results of an analysis of Dutch primary healthcare records demonstrate a significantly lower prescribing rate compared with the UK, with ∼22.5% patients consulting with a RTI episode, being issued a prescription.^11^ Since majority of antibiotic prescribing takes place in primary care, the management of these infections offers an opportunity to make a major impact on unnecessary antibiotic prescribing. The Department of Health as a part of the UK Antimicrobial Resistance Strategy identified education and training as a key measure to reduce inappropriate and unnecessary antibiotic prescribing.^12^

A number of previous randomised controlled trials have tested strategies to reduce unnecessary antibiotic prescribing. A review of such interventions conducted up to 2007 which included 30 trials, found a median reduction in the proportion of participants receiving antibiotics of 9.7% (IQR 6.6–13.7%).^13^ Most studies employed educational activities aimed at clinicians or patients, or audit of antibiotic prescribing with feedback of results, or a combination of these interventions. Recent trials which have used similar intervention strategies, but have more frequently used electronic media to deliver advice on appropriate prescribing,^14^ ^15^ have demonstrated similar reductions in antibiotic usage, with reduction in antibiotic prescribing of up to 15% in the GRACE trial. However, previous trials required resource-intensive interventions, and these intervention techniques have not yet been translated on a wide and sustainable scale into routine healthcare. For example, the trial by Gonzales *et al*^16^ required clinicians to participate in a half-day training session, with triage nurses providing patients with education leaflets to read before their consultation. The challenge now is to take the components of intervention that have been shown to be effective and to find methods to deploy these efficiently into routine practice settings.

Our group recently completed a trial (electronic Cluster Randomised Trial, eCRT) in which 104 general practices in England and Scotland, which contributed electronic health records (EHRs) to a national primary care database the CPRD, were randomised.^17^ Intervention practices had decision support tools delivered remotely using the practice systems that are employed in delivering routine primary care. These decision support tools on antibiotic prescribing appeared on intervention family practitioners' screen during consultations for specific RTIs. This simple intervention showed a near two percentage point reduction in antibiotic prescribing.^18^ This trial also demonstrated that EHRs can be used successfully as a means to inform, deliver and evaluate the effectiveness of an intervention to support reduced antibiotic prescribing.

Process evaluation undertaken as a part of the eCRT study suggested that although the intervention resulted in a significant reduction of antibiotic prescriptions among intervention practices, the intervention tools have been underused by many participating general practitioners (GPs). For example, some GPs were not aware of the implementation of the system into their practice.^16^ These findings taken together with evidence from the systematic review, recent trials and systematic reviews of the wider implementation science literature,^19^ ^20^ identify ways to increase engagement in the intervention and increase effect size.^21^ This research is at a later stage of translation than previous randomised trials evaluating strategies to reduce antibiotic prescribing. In order to overcome the block in the translational pathway, there is now a need to develop and evaluate more effective complex multicomponent interventions that can be implemented and delivered remotely. The research will focus on interventions that can be readily scaled up, through remote delivery using electronic media, to large samples of unselected practices.

## Aims

The primary objective of the proposed study is to evaluate whether a complex multicomponent, but low-cost intervention to influence GPs' prescribing of antibiotics when patients consult with RTIs, delivered electronically at the level of general practice, reduces antibiotic prescribing rates in primary care.

## Methods/design

This trial is a two-arm cluster randomised trial with general practices as the unit of allocation. Consenting GP practices who meet eligibility criteria (as defined in Study setting and target population section) are allocated to intervention and control trial arms by minimisation. Control trial arm practices will continue with usual clinical care. Usual care has been chosen as the control groups since current trail aims to test if current intervention is better than or at least equivalent to current clinical practice. An internal pilot will be conducted to demonstrate the feasibility and acceptability of the intervention in 20 general practices. The components of the intervention will be delivered to practices allocated to the intervention trial arm. In the pilot phase, intermediate outcome measures will include (1) successful installation of the decision support tools at intervention practices; (2) successful delivery of practice prescribing reports and webinars to intervention trial arm practices; and (3) evidence that the intervention tools are accessed and used by prescribing members of staff at intervention trial arm practices. Components of the trial that are deemed to be unacceptable or unfeasible will be modified. The remaining practices will be allocated once there is evidence that the interventions are being successfully delivered and used by practices.

### Study setting and target population

The study will be conducted in the CPRD. The CPRD is the largest primary care database of longitudinal medical records worldwide and includes about 7% (coverage of over 11.3 million patients) of the UK general practices.^22^ The CPRD data are generated via GP computer systems, and special software collects data from practice servers on a monthly basis. The CPRD collect anonymised data on clinical diagnosis, laboratory tests, issued prescriptions, clinical referrals and hospital admissions. To record healthcare, GPs can use a combination of coded and free-text data.^22^ The registered population is generally representative of the UK general population in terms of sex, age and ethnicity, and the quality of EHRs data in the CPRD are well described.^23^ General practices in England, Scotland, Wales and Northern Ireland that presently contribute research quality data to the CPRD will be eligible for the study.

General practices which contribute data to CPRD will be invited to participate by CPRD and will be asked to provide written consent. Only those practices that use DXS-Point-Of-Care software, Vision system software and which are located in areas that have given research governance approval for the study will be eligible to participate. The target population for this trial is the general population registered with general practices in the UK, including England, Scotland, Wales and Northern Ireland. The immediate participants in the research are health professionals who may issue prescriptions for antibiotics at the UK general practices.^24^ Outcomes will be evaluated using the anonymised EHRs for individual patients registered with the UK general practices who may consult with RTIs and receive antibiotic prescriptions.

### Ethical approval

CPRD general practices that give informed consent to the study will be included in the trial. The intervention is at general practice level; therefore, individual patient consent will not be sought.

### Sample size calculations

Stata V.13 was used for calculations. In order to obtain a result as precise as possible, we aimed to achieve the maximum feasible sample size. At the trial start in January 2015, there were 427 general practices active in CPRD. Based on previous experience,^18^ we estimated that it would be feasible to recruit a maximum of 120 CPRD general practices. The mean practice list size was 8537, and 120 general practices will include some 1.20 million registered patients, with about 224 000 RTI consultations over 12 months. Power calculations were computed based on primary outcome of antibiotic prescribing for RTI per 1000 participant-years, using data from the previous eCRT study.^18^ In the eCRT study, which included participants aged 18–59 years, the mean antibiotic prescribing rate for RTI was 112 per 1000 (SD 39.8). Therefore, for this study, based on the analysis of covariance, where participants of all ages will be included, including 60 GP practices in each trial arm, there will be more than 80% power, with two-sided α=0.05, to detect an absolute reduction in antibiotic prescription for RTI of 12 per 1000 registered patient years (or 1.2 per 100). If the SD is 25% larger, the study will still have 80% power to detect a reduction in antibiotic prescribing of 15 per 1000 (1.5 per 100 patients), or 17.5 per 1000 if the SD is 50% larger.

### Allocation

The allocation will be performed at King's College London using anonymised practice identifiers passed from CPRD. The research team are at all times blind to the identity of trial practices, which is only known to CPRD staff. GP practices are allocated to intervention and control trial arms by minimisation controlling for baseline antibiotic prescribing quartile region (England, Scotland, Wales and Northern Ireland). Anonymised practice identifiers will then be returned to CPRD with trial arm allocation attached. This information will then be used to enable intervention activation at practices in the intervention trial arms. This procedure is considered to ensure adequate concealment throughout the allocation process.

### Intervention development and implementation

The development of the intervention was informed by existing health records, behaviour change theory, systematic review evidence, clinical guidelines, qualitative research with non-trial practices (31 GPs and 3 nurse prescribers interviewed), as well as process evaluation data from the previous proof-of-concept trial.^25^ Main elements used in the previous eCRT study were refined and new elements were added. Two novel major components are the provision of practice-level feedback on antibiotic prescribing and recruitment of a GP champion for each practice since facilitation plays an important role affecting the context in which change occurs.^26^ A large part of the intervention refinement focused on the investigation of factors influencing implementation and selection of modifications of the tools in order to achieve maximum benefits of the intervention.

Practices in the intervention arm will receive complex multicomponent interventions, delivered remotely, which will include a 6 min web-based training webinar to promote effective usage of the intervention materials; prescribing support tools which will appear on intervention family practitioners' screens during consultations for specific RTI; monthly feedback on practice antibiotic prescribing in the preceding month from EHR analysis. Control practices will continue with usual care. The intervention will continue for 12 months. A detailed description of the development and design of the electronic prompts has been reported elsewhere^27^ and will be updated for this study.

In order to preserve practices' anonymity, general practice recruitment will be conducted through the offices of CPRD. To ensure that an adequate practice recruitment and enrolment is achieved, regular meetings will be held with CPRD and Trial Steering Committee (TSC) overlooking the recruitment process. All CPRD general practices that are located in areas where research governance approvals have been obtained will receive an invitation pack, including a letter, consent form and information sheet. CPRD general practices that give informed consent to the study will be included in the trial. Intervention tools will be installed onto family practice information systems remotely as an add-on to existing software (DXS-Point-Of-Care). At the beginning of the intervention, GPs and nurse prescribers at intervention practices will be encouraged to watch a 6 min video narrated by a practising GP which aims to present the study and promote its effective usage. Once the tools become available on the practice system, a pop-up banner would appear after the first log-in to inform the doctor/nurse that the study tools are available on their system. During consultations for RTIs, electronic prompts will be activated by the entry of specific Read codes related to RTIs. Healthcare professionals will see the prompts in the right bottom corner of their screen and these prompts would offer two options to select: an option to print out a patient information leaflet or an option to check whether a patient is among a group of patients who are likely to be at risk of developing complications. Five condition-specific leaflets are available for adults (for common cold, sore throat, cough and bronchitis, sinusitis and otitis media). There are also two separate leaflets for parents of children who present with cough or middle ear infection. The leaflets give patients information on realistic recovery times, self-management strategies, explain why antibiotics are not needed, inform about the modest benefits and potential harms from antibiotic treatment, list serious signs of when patients should seek medical help and provide patients with clear guidelines as to when they should reconsult if their symptoms do not improve. These prompts aim to help GPs follow the guideline behaviour. The main focus is to encourage GPs not to prescribe antibiotics rather than to prescribe an antibiotic or offer a delayed prescription. All management decisions for individual patients remain at the discretion of individual GPs. Each practice in the intervention arm will also receive monthly feedback on their antibiotic prescribing in the preceding month from CPRD analysis. The reports would present the prescribing rates in a table format and would also include evidence for safe best practice in RTIs management. Practices will be encouraged to review the monthly feedback received as part of the trial during practice meetings. GP champion for each practice will be encouraged to circulate the feedback prior to the meeting and ensure that the discussion of the feedback is on the meeting agenda.

### Outcome evaluation

The effectiveness of the intervention will be evaluated by analysing EHRs that are routinely collected into the CPRD database during a defined study period and historical information will be used to assess preintervention data. Data available for each participant will comprise their entire anonymised electronic medical record, including medical (Read) codes associated with consultations and referrals, and details of all drugs prescribed.^22^ CPRD data are also linked to Hospital Episode Statistics (HES) data for consenting practices in England. The primary outcome will be the number of antibiotic prescriptions for RTI per 1000 patient years. Secondary outcomes will be: the RTI consultation rate; the proportion of consultations for RTI with an antibiotic prescribed; subgroups of age; different categories of respiratory infections, including colds, sore throat, cough and bronchitis, otitis media and rhinosinusitis; and quartiles of intervention usage.

Analyses will be implemented according to the ‘intention-to-treat’ principle, including in the analysis all eligible person-time for all allocated practices, including data for any practices that later withdraw from CPRD or participants who subsequently ended their registration during the study period. Individual patient data will be included for participants who are currently registered with participating CPRD practices (no patient exclusion criteria). Preintervention data on antibiotic prescribing for the 12 months preceding the intervention will be analysed as baseline.

Trial analyses will be implemented using data aggregated to general practice level, using the family practice-specific rates or proportions as observations. This is the level for intended inferences. Effects of clinical and public health importance will be evident at this level. In general, a perfectly weighted cluster-level analysis will give similar precision as an individual-level analysis.^28^ Analyses for primary and secondary outcomes will estimate the difference (95% CI) in the outcome between intervention and control trial arms. Primary and secondary analyses will be adjusted for the preintervention value of the outcome, in an analysis of covariance framework, as well as proportion by age group and proportion of women at the practice. Minimum variance weights will be used to allow for varying numbers of participants and consultations per practice.^29^ Intervention usage (number of times prescribing reports or decision support tools are accessed) will be divided into quartiles and a trend tests implemented by introducing these into analyses as continuous variables. Data for healthcare usage and costs will be analysed at the individual level using a two-part model as reported previously.^30^ Given the extent of data available for analysis, we can readily evaluate shifts in practices' use of diagnostic categories, using pretrial data to evaluate time trends.

### Process evaluation

A process evaluation will be conducted to evaluate the barriers and facilitators to implementation and the use of the intervention using a mixed-methods approach. Participants in the process evaluation will primarily include GPs, but staff involved with intervention implementation will also be included, aiming pragmatically for the maximum achievable sample. We will aim to recruit practitioners with a range of experiences of the intervention to explore their unique and important perspective. A questionnaire and an interview guide will be developed guided by criteria suggested by Linnan and Steckler^31^ for the process evaluation of public health interventions and research and will explore participants' experiences of using the intervention materials and experiences of the study implementation. Inductive thematic analysis will be used to analyse qualitative data. As a part of process evaluation, contextual information on initiatives to influence antibiotic prescribing, which might be implemented both locally and nationally, will be collected. This will include periodic surveys of documentary sources, primarily those accessible on the internet. It will also include specific questionnaire items concerning participating practices' exposure to other influences, such as interaction with the local National Health Service (NHS) prescribing advisers. As a part of process evaluation, compliance with the intervention protocol will be assessed. This will be done by evaluating the total number of times the intervention tools (including the practice prescribing reports, the decision support tools and webinars) are accessed over the intervention period.

#### Participant safety

Safety outcomes would include diagnoses of pneumonia, empyema, peritonsillar abscess, mastoiditis, intracranial abscess, meningitis, osteomyelitis, pyelonephritis, Scarlet fever, septic arthritis, septicaemia/toxic shock and mortality. The incidence of these will be compared between trial arms and across high and low antibiotic prescribing practices divided into quartiles.

#### Adverse events/reactions

All management decisions for individual patients remain at the discretion of individual GPs. Therefore, we do not anticipate any potential serious adverse events that could be directly attributable to the intervention. However, we will ask GPs at participating general practices to notify the study team of any possible adverse events. If any such reports are received the TSC and the Research Ethics Committee will be notified.

#### Independent monitoring and quality control

TSC including a Data Monitoring Committee (DMC) have been set up to monitor the conduct of the trial and will meet throughout the study duration. The TSC will include among others a member of the patient participation group and an independent GP member.

### Economic evaluation plan

Total healthcare usage costs will be estimated from CPRD data, using methods reported previously,^18^ and compared between trial arms. Analyses will evaluate primary care usage including consultations at the practice, emergency consultations, home visits, out-of-hours visits and telephone consultations; hospital usage included inpatient admissions, outpatient episodes, day cases and emergency episodes. A standard two-stage approach will be used to estimate costs. A probit model will be used to estimate the probability of any healthcare being used, because some patients will not use healthcare during the period of study. A general linear model will then be used to estimate the mean costs for participants who make any use of healthcare. The costs of healthcare will be multiplied by the probability of using healthcare to obtain the final estimate, which will be compared between trial arms.

#### Reporting and dissemination

A number of publications in peer-reviewed journals are expected from this trial and these will include description of the intervention development and intervention content; main findings of the trial; findings of a mixed-methods evaluation of trial procedures. All these publications will be made available in open access journals in order to provide access to all readers anywhere in the world.

#### Protocol amendments

Protocol amendments will be communicated to the Study Management Group, the TSC, the DMC, the funder (National Institute for Health Research (NIHR) Health Technology Assessment programme) and to the Research Ethics Committee.

#### Study status

The intervention development phase has been completed. Recruitment for the trail started in August 2015. The first batch of 19 practices was randomised in November 2015 and these practices acted as an internal pilot. Currently, 76 practices are part of the study.

## Discussion

This intervention will use EHRs as a means to inform, deliver and evaluate the effectiveness of a cluster trial to support antibiotic prescribing. The 60 trial practices which will be randomised to the intervention arm of this trial may include more than 600 000 individual participants, allowing detection of small effects that could be widely implemented and be of public health importance. Careful planning of this intervention could help to overcome some of the challenges associated with deploying effective intervention components into routine practice setting. In addition, this trial will provide evidence on more effective roll-out of strategies at changing prescribers' behaviour into routine practice settings without resource-intensive interventions. A step-wedge design might be considered in evaluating the future roll-out of apparently successful interventions.^32^ A key output from this research will be establishing a way of delivering a multicomponent intervention through electronic media in order to change antibiotic prescribing behaviour in primary care. Importantly, rigorous process evaluation conducted as a part of this study will examine facilitators, barriers and obstacles to implementation of this intervention and assess compliance with the intervention protocol. This will help to establish whether the behaviour of health professionals was modified as a result of being part of the study or being exposed to the intervention tools. If effective, the intervention could be easily translated into routine practice settings at very low cost.
